# On the Present State of Medicine in the United States

**Published:** 1837-01

**Authors:** Robley Dunglison

**Affiliations:** Professor of the Institutes of Medicine and Medical Jurisprudence in Jefferson College, Philadelphia


### PART FIFTH.

### Medical Intelligence.

ON THE

### PRESENT STATE OF MEDICINE IN THE UNITED STATES;

By Robley Dunglison, m.d., Professor of the Institutes of Medicine and Medical Jurisprudence in Jefferson College, Philadelphia.

### ART. I. OF THE MEDICAL INSTITUTIONS, (continued.)

#### B. MEDICAL ASSOCIATIONS FOR THE REGULATION OF PRACTICE.

Although the colleges which possess the power of conferring degrees are so numerous in the United States, they are not the only institutious that give authority to practice. In some of the States, the diploma of an incorporated Institution is of itself insufficient; the Candidate for the exercise of his profession must still subject himself to examination before a State Medical Society, and not until he has passed this Ordeal can he establish himself as a practitioner within the commonwealth.

Between fifty and sixty years ago, the first society was formed for the regulation of the practice of physic and the suppression of quackery. In 1781, the Massachusetts Medical Society was incorporated by the legislature of Massachusetts; and subsequently similar societies were instituted in Maine, New Hampshire, Vermont, Rhode Island, Connecticut, New York, New Jersey, Delaware, Maryland, Virginia, South Carolina, Georgia, Mississippi, Alabama, Louisiana, Ohio, Indiana, Illinois, the district of Columbia; &c. The main objects of all these associations are the same, but they differ essentially as to the powers which they possess. In some of the States, as in Virginia and Pennsylvania, persons who are not licensed, nay, who have no diplomas, are permitted to practise, and are allowed to receive the fees; whilst in other States, as in Massachusetts and Maryland, the unlicensed practitioner not only cannot recover, but he is subjected to a prosecution and to heavy penalties.

To appear before the examiners appointed by the society, it is not necessary that the candidate for the licentiate shall be a graduate of any Medical College. The censors have the power of granting licences to any one who exhibits himself, on examination, sufficiently qualified to exercise his calling. The act of incorporation of the Massachusetts' Medical Society, after stating that "it is clearly of importance that a just discrimination should be made between such as are duly educated and properly qualified for the duties of their profession, and those who may ignorantly and wickedly administer medicine, whereby the health and lives of many valuable individuals may be endangered, or perhaps lost to the community," enacts, that "the president and fellows of said society, or such other of their officers or fellows as they shall appoint, shall have full power and authority to examine all candidates for the practice of physic and surgery, who shall offer themselves for examination, respecting their skill in their profession; and if, upon such examination, the said candidates shall be found skilled in their profession, and fitted for the practice of it, they shall receive the approbation of the society, in letters testimonial of such examination, under the seal of the said society, signed by the president, or such other person or persons as shall be appointed for that purpose." It was subsequently, in 1819, enacted by the Senate and House of Representatives of Massachusetts, in general Court assembled, and by the Authority of the same, that no person entering on the practice of physic or surgery, after the 1st day of July of that year, shall be entitled to the benefit of law for the recovery of any debt or fee accruing for his professional services, unless he had, previously to rendering those services, been licensed by the officers of the Massachusetts Medical Society, or "*had been graduated a Doctor of Medicine in Harvard University.*" By this act, consequently, the graduate of any other Medical College in the State or elsewhere must present himself to be received as one of the *Permissi* of the society; the graduate of Harvard being alone exempt.

The following extracts from the By-Laws of that Society will shew, that the examination is not a mere matter of form.

"xxxiv. Any person, who has received his medical education within this commonwealth (Massachusetts), may be admitted to an examination by any board of Censors, provided he have the following qualifications, and not otherwise.

"1. He shall be a person of sound mind and of good moral character, shall have completed twenty-one years of age, and shall have such an acquaintance with the Latin language as is necessary for a medical and surgical education, and with the principles of geometry and experimental philosophy.*

"2. He shall have studied three full years under the direction, and attended the practice, of some one or more of the fellows, or retired or honorary members of the society; during which time he shall have studied the most approved authors on Anatomy, Chemistry, Materia Medica, Midwifery, and the theory and practice of Medicine and Surgery; or, at least, all those which the counsellors shall from time to time specify as constituting a proper course of medical and surgical education.

"xxxv. Any person, who has received his medical education out of this commonwealth may be admitted to an examination by any board of Censors, provided he have the following qualifications, and not otherwise:

"1. He shall have the qualifications first specified in the preceding by-law.

"2. He shall have studied three full years under the direction, and attended the practice of some respectable physician or physicians; and shall have followed in his medical studies a course equivalent to that pointed out in the second of the preceding by-laws."

This Society had not only the power of electing, from time to time, such physicians as are deemed worthy of membership, but of expelling those who may be found unworthy; a privilege, which they exercise on befitting occasions. Originally, the Society consisted of thirty-one members, but it now includes nearly seven hundred. On the day of the annual meeting of the fellows of the Society a dinner is provided, under the direction of the president, at the expense of the Society, for such fellows and professional strangers as may attend the meeting. It need scarcely be said, that in this way harmony is promoted amongst the members of the profession, and its respectability carefully protected. At the late anniversary meeting not fewer than 250 guests sat down under the presidency of their estimable officer, Dr. John B. Warren, of Boston, the able professor of anatomy in Harvard University.

At every annual meeting of the Society, as many "Counsellors" are chosen by ballot as the Society may consider at the time necessary and expedient, who hold their offices for one year. Amongst other matters, it is the duty of the Counsellors, once in every three years at least, to specify such authors on Anatomy, Physiology, Chemistry, Materia Medica, Midwifery, and the theory and practice of Medicine and Surgery, as they may judge proper and necessary to be studied by medical and surgical pupils, previously to, and to qualify them for, an examination before the Censors: and they are required to cause this specification to be published in three newspapers, in three different counties, and in at least two papers in the city of Boston. As the ballot may be presumed to fall generally on the older members of the profession, who are perhaps the

* "It is understood that he be able to translate the select orations of Cicero, the Æneid of Virgil, or the medical writings of Celsus, and the formulas of the Pharmacopœia of the United States; and that he have a knowledge of Euclid's, Playfair's, or Legendre's Elements of Geometry, and Bryan's Conversations on Experimental Philosophy, or Enfield's Elements of Natural Philosophy."

least likely to keep up with the advancing state of the science, the list of authors can scarcely be expected to be, in every instance, the best possible. In the specification last published, many of the best works are omitted, whilst others of but little importance or value have been retained.

The power given to the Society over its fellows is ample, and is occasionally exercised.

"Any fellow may be expelled from the Society, or, having resigned his fellowship, may be deprived of his privileges, by a vote of two-thirds of the fellows present at any annual meeting, upon charges of the following description; provided the charge or charges against him have first been considered by the counsellors, and are brought forward by them, or been made before the Society at a preceding annual meeting, and provided he has had opportunity given him to lay before the Society a refutation of the charge or charges so made, or a defence of his conduct in the premises, viz.

1st. For any gross and notorious immorality, or injurious crime under the laws of the land.

2. For any attempt to overturn or destroy the Society.

3. For the breach of any by-laws of the Society, for which expulsion is made the penalty.

4. For furnishing to any person a certificate in respect to his character and studies as a student of medicine, if the same be proved to be false, and shall tend to deceive the public or the Censors of this Society."

Another by-law declares that any person who is engaged in the practice of medicine or surgery in the commonwealth of Massachusetts, not being a fellow or licentiate of the Society, nor a doctor of medicine of Harvard University, shall be deemed an irregular practitioner; also any one who has been expelled from the Society, or who, after having been permitted to resign his fellowship, has been deprived of his privileges, or who has withdrawn himself from the Society without the permission of the Counsellors, shall be deemed by the fellows of the Society an irregular practitioner; and it is declared to be unlawful for any fellow of the Society, in his professional capacity, to advise or consult with any such irregular practitioner, or in any way to abet or assist him as a practitioner of medicine or surgery; and for any breach of this by-law the fellow of the Society is disqualified for one year from giving his vote at any meeting of the Society, and of the district society of which he may be a member; he is also liable to censure and reprimand from the counsellors, and, in aggravated cases, to expulsion. It is further declared, that if any fellow of the Society shall publicly advertise for sale any medicine, the composition of which he keeps a secret, or, in like manner, offers to cure any disease by any such secret medicine, he shall be liable to expulsion, or to such other penalty as the Society, at their annual meeting, may think proper to inflict.

The system has been found to work well; and hence the ranks of the profession are not better supplied in any State of the Union; nor its respectability better supported than in Massachusetts.

The fee charged by the Society for a licence is ten dollars.

Similar regulations to those adopted by the legislature of Massachusetts exist in some of the other States. In Maryland, the association incorporated by the legislature is termed the "Medical and Chirurgical Faculty of Maryland." The first act to establish it was passed in 1799, empowering the Faculty "to grant licences to such medical and chirurgical gentlemen as they, *either upon a full examination, or upon the production of diplomas from some respectable college*, might judge adequate to commence the practice of the medical and chirurgical arts; each person so obtaining a certificate, to pay a sum not exceeding ten dollars, to be fixed on or ascertained by the Faculty. The act farther declared, "that, after the appointment of the Medical Board, no person not already a practitioner of medicine or surgery should be allowed to practise in either of the said branches, and receive payment for his services, without having first obtained a licence, certified as by this law directed, under the penalty of

*fifty dollars* for each offence, to be recovered in the County Court, where he may reside, by bill of presentment and indictment; one-half for the use of the Faculty, and the other for that of the informer." In one of the by-laws of the Faculty it is enacted, that "even persons obtaining a licence from the Board of Examiners to commence the practice of medicine and surgery, shall pay into the Treasury the sum of ten dollars. And all persons, graduates or others, wishing a licence to practise medicine and surgery in Maryland, are equally bound to pay for said licence." It has been decided, however, of late, by the Court of Appeals of the State, that a diploma from the University of Maryland is a State licence, and, therefore, her Alumni are exempt from the demand.

The annual convention of the Faculty takes place on the first Monday of June. Its object is to investigate and regulate the concerns of the Faculty; for which occasion an orator is appointed to address the Faculty on some medical or philosophical subject. The meetings are not, however, attended with the same zeal as those of the Massachusetts Medical Society, the business concerns of the Faculty being generally left to the management of but a few members. A portion of the funds accruing to the Faculty is appropriated, as in the case of the Massachusetts Medical Society, to the formation of a medical library, which is in Baltimore, and to which the members have access. It has not been long founded; but it already possesses upwards of six hundred volumes, the productions of some of the best authors in the different departments of the science.

The formation of these State Societies has doubtless had considerable influence in promoting the respectability of the profession, and harmony amongst its members, and, to a certain extent, in preventing quackery. Still, even in States of the Union in which such societies exist, empiricism is too much tolerated. Although inhibitory laws may have been enacted, it is not easy to have them enforced; indeed sympathy is often enlisted in behalf of the defendant when a prosecution is undertaken, and mainly on the ground that it is an interference with the just rights of the citizen, who ought to be permitted to exercise any calling he may select; and that, if the public choose to consult an unqualified individual, they should have the right to do so; that, moreover, it is creating a monopoly in the case of the regular practitioners, which ought not to be countenanced. These reasons have at times had sufficient weight in legislative bodies to induce a majority of the members to vote for the admission of the unlicensed pretender to full rights. Not long ago, an application was made by a class of empirics, existing everywhere in the United States, who call themselves Thomsonians after their founder, to the legislature of Maryland, to permit them to establish an Infirmary within the State, and to teach medicine uncontrolled by the Medical and Chirurgical Faculty; but the good sense of the legislature prevailed, and the application was rejected.

The Thomsonians consider that most diseases are owing to defect of stimulus, and accordingly they administer largely the different peppers, especially the cayenne, and stimulate by the vapour bath. They discard all mineral remedies, and profess to be essentially (as they term themselves) Botanic Physicians. The number of their proselytes in the United States amongst the unprofessional is considerable. Perhaps the best way to check its spread is to trust to the good sense of the community. Like every other form of empiricism, it may exist for a time, and they who succeed us will be surprised at our gullibility, as we are surprised at the gullibility of those who have gone before us.

By several of the societies of the different States, systems of medical ethics have been drawn up for the guidance of their members, containing such regulations of etiquette as are calculated to maintain their honour and dignity, and to keep up a good feeling amongst the members. In the year 1832, the Medico-Chirurgical Society of Baltimore published a system of medical ethics adopted by them on the report of a committee. In the composition of this system the Committee affirm that free use was made of Percival's ethics, the abridgment of the same by the Kappa-Lambda Society of Philadelphia, Gregory's Lectures on the Duties and Qualifications of a Physician, the Code of Ethics drawn up by the New-York State Medical Society, that of the Connecticut Medical Society, Rush's Medical Observations and Inquiries and Lectures, and the Medical Jurisprudence edited by Dr. Griffith, of Philadelphia. The first section embraces the Duties of Physicians to each other. Several of these appear to be sufficiently obvious, and their very mention might seem to be a work of supererogation. Were the great rule of Christian ethics present to the mind of the physician, "Do unto others as ye would that they should do unto you," there would be but little necessity for such a publication. The following are some of the printed inculcations.

"1. Every physician should observe the strictest caution and decorum in his intercourse with a case under the care of another; as the mind of the patient, as well as that of the friends, is, by solicitude and anxiety, rendered peculiarly sensible to every thing relating to the management and termination of the case, and is ever ready to seize upon every meddling enquiry and every disingenuous hint, and to construe them in a manner disadvantageous to the physician employed, and calculated to destroy the confidence reposed in him.

"2. The same caution and circumspection should be observed when, from motives of business or friendship, a physician is prompted to visit an individual who is under the direction of another practitioner. Indeed, such visits should be avoided, except under particular circumstances; and, when they are made, no particular enquiries should be instituted relative to the nature of the disease or the remedies employed; but the topics of conversation should be as foreign to the case as circumstances will admit. It is also indecorous and undignified for a physician to visit an individual, or in families one of whom may be sick, with a view of seeking employment, as he thereby evinces a meanness of disposition and a desire to obtrude his services, where a proper confidence may not be reposed in his skill and judgment.

"4. In cases of accident or emergency from other causes, a physician is frequently summoned to visit a patient who is under the charge of another, to whom, in consequence of his being out of the way, notice of such call has not been given. It will be proper, under such circumstances, for the physician last called to resort to such remedies as the urgency of the case may require; but, if the condition of the patient will admit of delay, no alteration of the treatment should be made without a previous consultation with the attending physician.

"6. As there may be an honest difference of opinion amongst medical men with regard to the nature and treatment of diseases, it should be deemed dishonorable, and a violation of medical ethics, for one physician to charge another with malpractice, or to throw out any insinuation that he has treated a case improperly, except such accusation be made before legitimate judges, or such persons as are properly empowered or constituted to hear and investigate the circumstances of the case.

"The situation of a physician, the character of his profession, and the nature of his intercourse with the sick, must of necessity render him, to a certain extent, the confidant of families, and familiar with the foibles and infirmities of individuals. He should therefore regard it as a sacred obligation to conduct himself with the most scrupulous regard to secrecy; to maintain the most honest and chaste observance of the confidence reposed in him, and never to divulge, except when compelled, in obedience to the laws of the country, the nature of the malady he is called upon to treat, the private affairs of families or individuals, the faults or infirmities which may fall under his observation, or any circumstance that could tend to wound the feelings or stigmatize the character or reputation of those whose confidence he enjoys. The force and necessity of this obligation are indeed so great, that professional men have, under particular circumstances, been protected in their observance of secrecy, even by courts of justice.

"11. Every individual, on entering the profession, as he becomes thereby entitled to all its privileges and immunities, incurs an obligation to exert his best abilities, to maintain its dignity and honour, to exalt its standing, and extend the bounds of its usefulness. He should therefore observe strictly such laws as are instituted for the government of its members; should avoid all contumelious and sarcastic remarks relating to the faculty as a body; and while, by unwearied diligence, he resorts to every honorable means of enriching the science, he should entertain a due respect for his seniors, who have by their labours brought it to the honorable and elevated condition in which he finds it."

Such are a few of the rules laid down as regards the "duties of physicians to each other."

In the section on the Duties of the Faculty in relation to quackery, the profession in Baltimore do not accord with their brethren in Great Britain, on the subject of offering advice to the poor, gratis. "The honour and importance of the profession," say they, "render it incumbent on its members to maintain its respectability, and avoid every act which could compromise its dignity, or forfeit the high respect and confidence reposed in it. He, therefore, who resorts to unapproved methods of practice, dispenses secret nostrums, or employs intrigue and artifice to secure business, degrades the medical character, and lowers himself to the level of a mere quack. It is also derogatory to the dignity of the profession, and constitutes a species of quackery, to resort to public advertisements or private cards or handbills, inviting the attention of individuals affected with particular diseases, publicly "offering advice and medicine to the poor, gratis," promising radical cures, and stipulating for "*no cure no pay*," publishing cases and operations in the daily prints, or suffering such publications to be made; boasting of cures and remedies; adducing certificates of skill and success, or any other similar acts which are commonly resorted to by known quacks and gross pretenders."

Of the rules of "Conduct to be observed in relation to Consultations," the following are of universal application.

"A physician who is called upon to consult, should observe the most honorable and scrupulous regard for the character and standing of the gentleman in attendance: his practice, if necessary, should be justified as far as it can be, consistently with a conscientious regard for truth and honesty, and no hint or insinuation should be thrown out which could impair the confidence reposed in him or affect his reputation. He should also carefully refrain from any of those extraordinary attentions or assiduities which are too often practised by the dishonest for the base purpose of gaining applause or ingratiating themselves into the favour of families and individuals."

"As a regular medical education affords presumptive evidence of professional abilities and acquirements, and the only acknowledged right of an individual to the exercise and honours of the profession, no consultation should be held with any individual who has not complied with the laws enacted for the proper regulation of the practice of medicine in the State in which he resides, or who is not in possession of a diploma from some medical college or university of known and acknowledged respectability. Consultations should, under no pretext, be held with unqualified persons or quacks, or with such members of the profession as have by improper conduct outraged its dignity and respect."

If an attention to professional etiquette be important where the profession is divided, as in Great Britain, in such sort that the apothecary is the regular family attendant, whilst the physician or surgeon is called in only in consultation, an attention to it is obviously still more requisite where, as in the United States, there is but one class of practitioners, and where it is therefore easy for the consulting physician, who may not be overscrupulous, to supplant the family attendant. Hence it is that many of the State Societies have published codes of medical ethics for the guidance of their members.

### ART. II. OF THE REMUNERATION OF THE MEDICAL PROFESSION.

The fees for professional attendance differ somewhat in different cities and districts of the Union. For an ordinary visit made by the family attendant, the remuneration is much the same everywhere; and the same may be said of the consulting physician, his first visit entitling him to the sum charged in the following tables, and the subsequent visits being usually treble those of the primary attendant.

To guide the physician in making his charges for services rendered, and to prevent disputes on the part of the recipient of such services, &c. tables have been formed in many of the cities; of which those of New York, Baltimore, and Charleston are at this time before me. The following tabular view will exhibit the comparative rates. Those in the first table were established by associated physicians and surgeons of the city of New York, in December, 1815, and approved by the New York County Medical Society, in January, 1816: those in the second were adopted by the Medico-Chirurgical Society of Baltimore, and other members of the medical profession of that city, in 1832: and those in the third by the members of the Medical Society of South Carolina, in the years 1792, 1804, 1813, and 1821.

A. *Comparative Table of the Medical, Surgical, and others Fees, of New York, Baltimore, and Charleston.*

| | New York. | Baltimore. | Charleston. |
|---|---|---|---|
| | | | £ s. d. |
| For the first visit . . | 0 to 2 dollars* | 1 to 2 dollars | 0 5 0† |
| Every subsequent visit . | 0 to 2 dollars | 1 ... | |
| For a single visit and advice in special cases . . | ......... | 2 to 10 ... | |
| First consultation visit . | 5 ... | 5 ... | 3 5 4 |
| Each ditto after the first . | 3 ... | 1 to 2 ... | 0 10 0 |
| Opinion in writing‡ . . | 10 to 15 ... | 5 to 10 ... | 1 to 3 guineas. |
| Advice at the physician's house, according to its importance | ......... | 1 to 10 ... | |
| Advice at the physician's house, at night§ . . . | ....... .. | 2 ... | 0 10 0‖ |
| Visiting at night§ . . | 7 ... | 5 ... | |
| Detention . . . | 3 doll. per hour<br>25 doll. per day | ......... | 0 10 0<br>per hour. |
| Detention in any case at patient's house during the night‖ . | ......... | 5 to 20 ... | |
| Visit at a distance, per mile; night visit double¶ . . | 1 50 c. | 1 ... | |
| First visit in epidemic or other diseases, where personal danger is apprehended . . | 5 ... | ......... | |
| Each succeeding visit, under the same circumstances . . | 3 ... | ......... | |
| For a requested visit after dark | ......... | ......... | 0 10 0 |
| For rising out of bed and visiting, according to the weather and other circumstances . . | ......... | ......... | £1 to £2 |
| Case of midwifery, natural . | 25 to 35 ... | 10 to 25 ... | 7 0 0 to<br>11 13 4 |
| Ditto, preternatural | 36 to 60 ... | 25 to 50 ... | 11 13 4 to<br>18 13 4 |

* Visits in haste charged double in New York.
† Currency 4*s.* 8*d.* to the dollar. ‡ By letter.
§ "The night is considered, in these cases, as beginning at ten o'clock P.M., and ending at sunrise."—*Baltimore.*
‖ After having gone to bed.—*Charleston.* ¶ Baltimore,

| | New York. | Baltimore. | Charleston. £ s. d. |
|---|---|---|---|
| Extracting placenta alone . | ......... | 10 to 25 dollars | |
| Visit and advice given to midwife, in cases of labour . . | ......... | ......... | 7 0 0 |
| Bleeding . . . | 2* dollars | 50 c. to 1 dollar | 0 9 4 |
| in the jugular vein . | 5 ... | ......... | 1 3 4 |
| Arteriotomy . . . | ......... | ......... | 1 3 4 |
| Extracting a tooth . . | 1† ... | 50 c. to 1 dollar | |
| Vaccination . . . | 5 to 10 ... | 2 to 5 dollars | 1 3 4 to 2 2 0 |
| Case of gonorrhœa or syphilis, (fee in advance) . . . | ......... | 10 to 50 ... | |
| Case of gonorrhœa . . | 15 to 30 ... | ......... | 3 10 0 to 7 0 0 |
| Case of syphilis . . | 25 to 100 ... | ......... | 3 10 0 to 23 6 8 |
| Amputation of large limbs . | 50 ... | 25 to 75 ... | 5*l.* to 7*l.* |
| of great joints . | 100 to 150 ... | 50 to 100 ... | 23 6 8 to 35 0 0 |
| of fingers and toes . | 10 ... | 5 to 20 ... | 1 0 0 |
| of ditto, through tarsal or metatarsal bones | ......... | 25 to 50 ... | 4 13 4 to 7 0 0 |
| Excision of diseased joints . | ......... | 50 to 100 ... | |
| Operation for necrosis and exostosis | ......... | 30 to 50 ... | 5 16 8 to 11 13 4 |
| of trepanning . | 100 ... | 30 to 50 | 10 0 0 |
| Reduction of dislocations . | 5 to 20 ... | 10 to 50 ... | 10*s.* to 14*l.*‡ |
| Adjustment of fractures of long bones | 10 to 30 ... | 10 to 30 ... | 1*l.* to 7*l.* |
| Lithotomy . . . | 150 ... | 50 to 200 ... | 10 0 0 |
| Reduction of hernia . . | 10 to 25 ... | 5 to 20 ... | 1*l.* to 5*l.* |
| Operation for strangulated hernia | 125 ... | 25 to 100 ... | 10*l.* to 15*l.* |
| Important operations on the eye | ......... | 20 to 100 ... | |
| Extirpation of the eye . . | 100 ... | ......... | 18 13 4 |
| Minor operations on the eye . | ......... | 5 to 20 ... | |
| Dressing recent wounds, opening abscesses, introducing seton or issue, &c. . . . . | 5 dollars, seton 2 dollars, issue | 1 to 10 ... | 1*s.* to 1*l.* |
| Each dressing, in addition to visit | 1 to 5 .. | 50 c. to 2 ... | Ditto |
| Extirpation of polypus . . | ......... | 5 to 30 ... | 7 0 0 |
| of tumours . . | 5 to 50 .. | 5 to 50 ... | |
| of tonsils . . | 25 ... | ......... | 7 0 0 |
| Operation for fistula in ano or in perineo . . . . | 50 ... | 10 to 50 ... | 4 13 4 to 10 0 0 |
| Passing catheter or bougie . | 5§ ... | 1 to 5 ... | 10*s.* to 1*l.* 10*s.* |
| Paracentesis of the thorax, abdomen, or bladder . . | 15 to 25, abdomen 50, thorax | 5 to 20 ... | 3*l.* abdomen and thorax, 4*l.* 13*s.* 4*d.* bladder |
| Operation for hydrocele . | ......... | 5 to 30 ... | 7*l.* to 10*l.* |
| for aneurism . | 25 to 200 ... | 25 to 100 ... | 5*l.* to 35*l.* |
| Ligature of arteries in cases of wounds | ......... | 25 to 50 ... | |
| Operation for harelip . . | 25 ... | 10 to 30 ... | 2*l.* to 5*l.* |
| Cupping . . . | 1 to 5 ... | ......... | 1 0 0 |
| Dressing blister . . | 1 ... | ......... | |
| Scarifying eye . . . | 5 ... | ......... | |
| Puncturing œdematous swellings | 2 ... | ......... | |
| Extracting calculus from the urethra | 20 to 30 ... | ......... | 4 13 4 |

* "In arm or foot."—*New York.*

† At the patient's house, two dollars.—*New York.*

‡ "Where the bone has been out of place for any considerable time, the charge for reduction is double."—*Charleston.*

§ The first time. Two dollars each succeeding time.—*New York.*

| | New York. | Baltimore. | Charleston. £ s. d. |
|---|---|---|---|
| Reducing prolapsus ani . | 5 dollars | ......... | 2 6 8 |
| Opening abscess . . | 1 to 5 ... | ......... | 5*s*. to 15*s*. |
| Amputation of the breast . | 50 ... | ......... | 10 0 0 |
| of the penis . | 20 ... | ......... | 11 13 4 |
| Extirpation of testis . . | 50 ... | ......... | 10 0 0 |
| Perforating rectum . . | 25 ... | ......... | 5 16 8 |
| nostrils, external ear, vagina, or urethra | 5 to 25 ... | ......... | 1 3 4 to 5 16 8 |
| Dividing frænum linguæ or of penis | 3 to 5 .... | ......... | 1 3 4 |
| Operation for phymosis . | 10 ... | ......... | |
| fistula lachrymalis | 40 ... | ......... | 10 0 0 |
| paraphymosis . | 10 ... | ......... | |
| cataract, by depression | 125 ... | ......... | 10 0 0 |
| by extraction | 150 ... | ......... | 10 0 0 |
| Bronchotomy . . . | 25 ... | ......... | 5 0 0 |
| Circumcision . . . | 10 ... | ......... | 2 6 8 |
| Preparing and administering enema | 2 ... | ......... | 1 3 4 |
| Extirpation of cancerous lip . | ......... | ......... | 3 0 0 |
| Opening sinuses . . | ......... | ......... | 1*l*. to 2*l*. |
| Extirpation of hemorrhoids . | ......... | ......... | 1*l*. to 5*l*. |
| Arresting hemorrhage . | ... ..... | ......... | 10*s*. to 3*l*. 10*s*. |
| Scarifying . . . | ......... | ......... | 0 10 0 |
| Electrical operations . . | ......... | ......... | 0 5 0 |
| The operation of sounding . | ......... | ......... | 10*s*. to 1*l*. 10*s*. |
| Puncturing the tympanum . | ....... .. | ......... | 3*l*. to 5*l*. |
| Inoculation . . . | ......... | ......... | 3 5 4 |
| Stricture of the urethra (case of) | ......... | ......... | 7*l*. to 15*l*. |
| Opening a lumbar or other large abscess . . . | ......... | ......... | 1 3 4 |
| Opening paronychia . . | ......... | ......... | 0 10 0 |
| Extracting foreign bodies from the œsophagus and other passages | ........ | ......... | 2 6 8 to 4 13 4 |
| Reducing prolapsus uteri . | ......... | ......... | 4 13 4 |
| Treating rupture of the tendo Achilles | ......... | ......... | 4 13 4 |
| Tying varicose veins of the leg & thigh | ......... | ......... | 4 13 4 |
| Extracting extraneous substances from gunshot and other wounds | ......... | ......... | 2 6 8 to 4 13 4 |
| Extracting foreign substances from the cavities of joints . | ......... | ......... | 4 13 4 |
| Operation for ranula . . | ......... | ... ..... | 2 6 8 |
| artificial pupil . | ......... | ......... | 23 6 8 |
| Application of bandages or adhesive straps . . . | ......... | ......... | 5*s*. to 15*s*. |
| Filling up a life-insurance policy | ......... | 5 to 20 dollars | |

Such are the standard fees of the cities in question, but for laudable—and too often perhaps for interested—motives, smaller fees than these are occasionally charged; a bargain is made between the medical adviser and the family, to attend them for so much *per annum*, but this is properly objected to in the Baltimore Fee Table, "as a measure unequal and often unjust in its action, on one or other of the parties, and as derogatory to the character and dignity of the medical profession in general." I have before remarked, that the fee per visit is much the same throughout the United States, both in the cities and in country situations. The "mileage," however, varies greatly. In the older settled States, not more than thirty-seven and a half cents per mile being charged; whilst, in the newer, to the south and west, the compensation is much the same as in the large cities,—a dollar per mile.

In Louisiana, Alabama, Mississippi, a fine field is offered for professional exertion. The proprietors of large cotton and sugar plantations require the constant attention of the physician, and remunerate him well. It is not unusual for the bill for professional services, on one of these estates, to amount to twelve or fifteen hundred dollars. Opportunities for investing money to advantage are perpetually occurring; so that the practitioner who is industrious and prudent speedily obtains enough to enable him to purchase a plantation, and to quit his arduous vocation: hence it is uncommon to meet with any but young practitioners in these situations. The great mass of those who are educated in the medical schools of Philadelphia and Baltimore, Lexington, and Cincinnati, seek the southern regions of their country; and, of late, medical colleges have been established in the south for the education of those whose circumstances or inclination do not permit them to repair to the more distant institutions.

In the larger cities of the Union, it is customary for the physician, as in Europe, to send his prescriptions to the apothecary, or *pharmacien*, to be compounded. Still there are many who furnish their own medicines, and in country situations such must necessarily be the case universally. In the cities the pharmaceutical, like the medical, charges have been established by the same Societies: such at least is the case with the Medical Societies of New York and South Carolina. The fee-table of the Medico-Chirurgical Society of Baltimore is defective in this respect.

B. *Charges for Medicines supplied by the Druggists.*

| | New York. dol. cen. | Charleston. £ s. d. |
|---|---|---|
| A single prescription furnished | 0 50 | ... ... |
| Pills, per dozen | 0 75 | ... ... |
| Boluses, each | 0 50 | ... ..., 0 3 0 |
| Electuaries, per ounce | 1 0 | ... ... 0 7 6 by the gallipot. |
| Infusions, per pound | 2 0 | ... ... |
| Solutions, per pound | 1 50 | ... ... |
| Tinctures, per ounce | 0 50 | ... ... |
| Ointments and cerates, per ounce | 0 50 | ... ... |
| Blistering plasters, according to size | 1 50 to 2 0 | ... 0 2 4 to 0 4 8 |
| Other plasters | 0 50 to 2 50 | ... |
| Decoctions, per pound | 2 0 | ... ... |
| A single medicine dispensed without visit | 1 0 | ... ... |
| An anodyne draught | 0 50 | ... ... |
| For an emetic or cathartic | .. | ... ... 0 1 6 |
| Mixtures, decoctions, or infusions, in phials, each | ... | ... ... 0 5 0 |
| Powders, according to their number and qualities, each, from 1s. to | ... | ... ... 0 1 6 |
| Pills, by the dose | ... | ... 0 1 0 |
| Draughts and other active medicines taken at once | | ... 0 3 0 |
| For all mixtures, decoctions, or infusions, containing from 1 oz. to 2 lbs. in phials, from 5s. to | | ... 0 15 0 |
| For materials for decoction or infusion, in papers, from 2s. 4d. to | | ... 0 9 4 |
| For active medicines taken by drops, in phials, from 5s. to | | ... 0 15 0 |

